# Baseline Assessment of Alcohol-Related Knowledge of and Support for Alcohol Warning Labels Among Alcohol Consumers in Northern Canada and Associations With Key Sociodemographic Characteristics

**DOI:** 10.15288/jsad.2020.81.238

**Published:** 2020-05-03

**Authors:** Kate Vallance, Tim Stockwell, Jinhui Zhao, Simran Shokar, Nour Schoueri-Mychasiw, David Hammond, Thomas K. Greenfield, Jonathan McGavock, Ashini Weerasinghe, Erin Hobin

**Affiliations:** ^a^ Canadian Institute for Substance Use Research, University of Victoria, Victoria, British Columbia, Canada; ^b^ Canadian Agency for Drugs and Technologies in Health, Toronto, Ontario, Canada; ^c^ Health Promotion, Chronic Disease and Injury Prevention, Public Health Ontario, Toronto, Ontario, Canada; ^d^ School of Public Health & Health Systems, University of Waterloo, Waterloo, Ontario, Canada; ^e^ Alcohol Research Group, Public Health Institute, Emeryville, California, United States; ^f^ Department of Pediatrics and Child Health, Faculty of Health Sciences, University of Manitoba, Winnipeg, Manitoba, Canada; ^g^ Dalla Lana School of Public Health, University of Toronto, Toronto, Ontario, Canada

## Abstract

**Objective:**

Evidence-informed alcohol warning labels (AWLs) are a promising, well-targeted strategy to increase consumer awareness of health risks. We assessed consumers’ baseline knowledge of alcohol-related cancer risk, standard drinks, and low-risk drinking guidelines as well as levels of support for AWLs. We further assessed associations with sociodemographic factors.

**Method:**

Forming part of a larger study testing new evidence-informed AWLs in a northern Canadian territory compared with a neighboring territory, baseline surveys were completed among liquor store patrons systematically selected in both sites. Chi-square and multivariable logistic regression analyses were performed to assess outcomes.

**Results:**

In total, 836 liquor store patrons (47.8% female) completed baseline surveys across both sites. Overall, there was low knowledge of alcohol-related cancer risk (24.5%), limited ability to calculate a standard drink (29.5%), and low knowledge of daily (49.5%) and weekly (48.2%) low-risk drinking guideline limits. There was moderate support for AWLs with a health warning (55.9%) and standard drink information (51.4%), and lower support for low-risk drinking guideline labels (38.7%). No sociodemographic characteristics were associated with cancer knowledge. Identifying as female and having adequate health literacy were associated with support for all three AWLs; high alcohol use was associated with not supporting standard drink (adjusted odds ratio = 0.60, 95% CI [0.40, 0.88]) and low-risk drinking guideline (adjusted odds ratio = 0.57, 95% CI [0.38, 0.87]) labels.

**Conclusions:**

Few consumers in this study had key alcohol-related health knowledge; however, there was moderate support for AWLs as a tool to raise awareness. Implementation of information-based interventions such as evidence-informed AWLs with health messages including alcohol-related cancer risk, standard drink information, and national drinking guidelines is warranted.

Alcohol is consumed by more than two billion people worldwide, with global estimates anticipating up to a 17% increase in consumption over the next decade (Manthey et al., 2019). The harms associated with its use are significant, and, in 2016, alcohol contributed to an estimated 3.3 million deaths globally (World Health Organization, 2018), accounting for approximately 4% of all cancer deaths (Global Burden of Disease 2016 Alcohol Collaborators, 2018). In Canada, 78% (22 million) of individuals age 15 years and older reported consuming alcohol in the previous year (Statistics Canada, 2018), and an estimated 15,000 people died of alcohol-attributable causes, one third related to cancer (Zhao et al., 2015). Despite these serious and significant harms, there is low knowledge of alcohol-related health harms, such as increased cancer risk, both internationally and in Canada. Alcohol is often perceived by the public to be less harmful than other controlled substances such as tobacco unless consumed in very high amounts (Buykx et al., 2015; Canadian Cancer Society, 2015; Pettigrew et al., 2016; Rehm et al., 2014; Rundle-Thiele at al., 2013; Wiseman & Klein, 2019), when in fact cancer risk increases even at low levels of alcohol consumption, particularly for breast cancer (Shield et al., 2016). This perception is of even greater consequence when considered in light of Canadian data where 69% of respondents indicated they would decrease their consumption levels if they knew that alcohol increased cancer risk (Canadian Cancer Society, 2015). International studies have found that greater knowledge of alcohol-related harms, particularly cancer risk, is associated with being female, older age, higher socioeconomic status, and adequate *health literacy*—defined as the ability to obtain and understand basic health information to make appropriate health decisions—as well as with lower levels of alcohol use (Buykx et al., 2015, 2016; Macdonald et al., 2011; Robb et al., 2009; Rundle-Thiele et al., 2013; Weiss, 2005).

Tools designed to inform alcohol consumers about minimizing alcohol-related health risks implemented in Canada and elsewhere include low-risk drinking guidelines (LRDG), which recommend daily and weekly consumption limits that are communicated using standard drink measurements (Butt et al., 2011; Kalinowski & Humphreys, 2016). However, to be effective, consumers must not only be aware of national drinking guidelines and the guidelines’ recommended limits but also understand how to apply them in relation to their own alcohol consumption, which is not often the case (Bowden et al., 2014; Bowring et al., 2012; De Visser & Birch, 2012; Livingston, 2012; McNally et al., 2019; Rosenberg et al., 2018). Similar to other countries with national drinking guidelines, only approximately one quarter of Canadian adults are aware that the LRDG exist, and more than 39% regularly drink in excess of the weekly and 27% in excess of the daily limits, after adjusting for underreporting (McNally et al., 2019; Statistics Canada, 2012; Zhao et al., 2015). Alcohol containers sold in Canada are mandated to list only percentage alcohol-by-volume information. Thus, the number of standard drinks (in Canada, one standard drink equals 13.45 g of pure alcohol), which is the unit of measurement used to convey Canada’s LRDG limits, is not provided on the alcohol container. The disconnect between the LRDG and the information currently listed on alcohol containers is likely one reason Canadian consumers are not aware of the LRDG and are unable to accurately monitor their alcohol consumption and comply with the recommended limits in the guidelines.

Presenting health messages on alcohol warning labels (AWLs) offers one relatively low-cost strategy to provide consumption information and to increase knowledge of alcohol-related risks, because heavier consumers are exposed to AWLs most often (Greenfield, 1997; World Health Organization, 2018). Previous experimental and laboratory based studies have indicated that not only did AWLs displaying standard drink information and LRDG limits improve consumers’ ability to estimate recommended consumption limits (Hobin et al., 2017; Osiowy et al., 2015), but adding labels with cancer warnings also decreased consumers’ motivation to drink (Blackwell et al., 2018). Recent real-world evidence using data from subsequent waves of the current study further showed that exposure to such labels increased knowledge of alcohol–cancer risk and daily and weekly LRDG limits, and reduced overall alcohol consumption over time (Hobin et al., 2020; Schoueri-Mychasiw et al., 2020; Weerasinghe et al., 2020; Zhao et al., 2020). Importantly, as knowledge of alcohol-related harms increases, so too does support for AWLs and other effective alcohol control measures shown to reduce alcohol harm, such as increasing minimum pricing and restricting alcohol availability and marketing; women, those who are older, and those who consume less alcohol are more likely to support such policies (Bates et al., 2018; Buykx et al., 2016; Li et al., 2017; Macdonald et al., 2011; Moskalewicz et al., 2013; Pechey et al., 2014; Weerasinghe et al., 2020). Unfortunately, the majority of labels implemented on alcohol containers globally to date do not communicate these types of messages, nor do they follow best practices for effective product label design, such as being larger, being prominently displayed on the container, having full-color graphics or images, and including personally relevant and direct messages that are regularly rotated to prevent wear-out effects (Ferrence et al., 2007; Fong, 2001; Hammond, 2011; Hobin et al., 2018; Vallance et al., 2018).

The current analysis forms part of a larger quasi-experimental study designed to test the real-world effectiveness of new evidence-informed AWLs presenting a cancer warning, national drinking guidelines, and standard drink information in two cities located in the northern Canadian territories, Whitehorse, Yukon, and Yellowknife, Northwest Territories. The aim of this article is to assess baseline levels of alcohol-related knowledge and of support for AWLs among consumers in the experimental sites, as well as associations with key sociodemographic and alcohol drinking factors. Specifically, this article investigates the degree to which participants know that alcohol can cause cancer, the number of standard drinks in an alcohol container, and the sex-specific daily and weekly standard drink limits recommended in Canada’s LRDG. The degree of support for labels on alcohol containers with a health warning, standard drink information, and Canada’s LRDG is also examined.

## Method

### Study design

**Figure 1.**
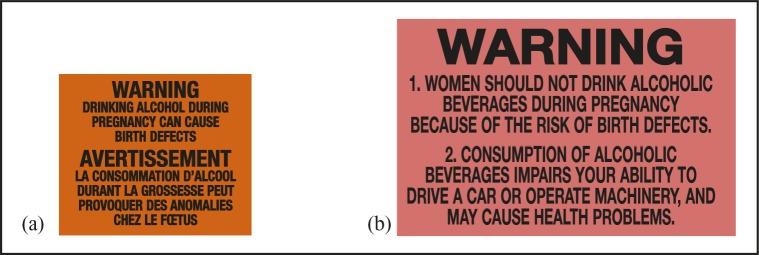
Alcohol warning labels on alcohol containers in (a) Yukon (2.3 cm × 2.8 cm) and (b) Northwest Territories (3.0 cm × 5.0 cm) since 1991

**Figure 2.**
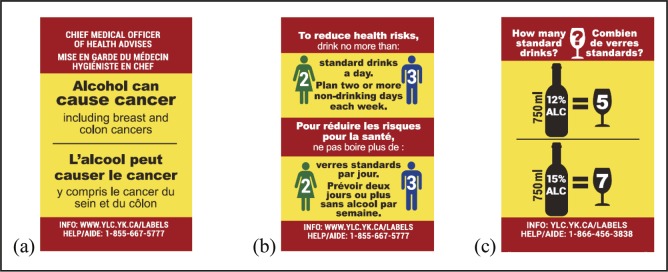
Intervention alcohol warning labels: (a) cancer warning, (b) low-risk drinking guidelines, and (c) standard drink information (5.0 cm × 3.0 cm)

Yukon and Northwest Territories are two northern Canadian territories with similar alcohol distribution systems, population size and demographics, and patterns of alcohol consumption and related harm (higher than the rest of Canada) (Canadian Institute for Health Information, 2017; Canadian Substance Use Costs and Harms Scientific Working Group, 2018; Statistics Canada, 2011a, 2011b, 2018). Further, these two territories are the only jurisdictions in Canada to apply after-market alcohol warning labels by local directive since 1991 (Figures 1a–b) and have well-established labeling procedures. A baseline survey was conducted with systematically selected liquor store patrons in the single government-run liquor store in the capital city of Whitehorse, Yukon (intervention site), and the two government-run stores in Yellowknife, Northwest Territories (comparison site), over a 6-week period in May and June 2017. The surveys formed part of a larger pre–post quasi-experimental study (see Vallance et al., 2020a, for full study protocol) testing the effectiveness of new enhanced evidence-informed AWLs with a cancer warning, national drinking guidelines, and standard drink information. The study was planned for an 8-month intervention in the one government-operated liquor store in the intervention site while usual labeling practices continued in the two government-operated liquor stores in the comparison site (Figures 2a–c).

Baseline survey participants were systematically recruited by trained research assistants (RAs) as they exited the liquor stores in the intervention and comparison sites, using a standard intercept technique of approaching every patron who passed a designated landmark. Surveys were administered on tablets in English and completed by participants independently without RA assistance. Up to two RAs recruited participants from Monday to Saturday (all stores are closed Sundays) between 10 A.M. and 6 P.M. in the intervention site and from 12 P.M. to 8 P.M. in the comparison site, covering comparable peak hours at both sites. Participants were screened for eligibility and had to be 19 years and older (legal drinking age in the two territories), had consumed alcohol in the previous month, had purchased alcohol at the liquor store the day of the initial recruitment, and did not self-report currently being pregnant or breastfeeding. Participants were offered a gift card in recognition of their time. The survey took an average of 18 minutes to complete, and participants were asked for email contact information to allow for email recruitment in subsequent waves of the study over the following year. Participants were recruited from the primary off-sale liquor stores in each city center in order to capture a broadly representative sample of adults purchasing alcohol in each site.

A total of 836 eligible participants were recruited and surveyed at baseline in the intervention (*n* = 505) and comparison sites (*n* = 331), with an overall response rate of 9.7% (American Association for Public Opinion Research, 2011). This response rate is consistent with other studies using a similar intercept technique (Hobin et al., 2017; Schneider, 2013; Wiggers et al., 2018). All recruitment and survey measures were consistent across the two sites. This study received ethics approval from the Research Ethics Board at Public Health Ontario (ID 2017-010.04) and the Human Research Ethics Board at the University of Victoria (Protocol 17-161), as well as the relevant research licenses required in Yukon and Northwest Territories.

### Measures

Survey measures were adapted from items used in evaluations of the AWL in the United States and Canada, in Canadian nutritional labeling practices, and in studies of tobacco warning labels (Greenfield & Kaskutas, 1998; Greenfield et al, 1999; Hammond, 2011; Hobin et al., 2018; Laughery et al., 1993; Pettigrew et al., 2016; Thomas, 2012).

### Knowledge that alcohol can cause cancer

To assess knowledge that alcohol can cause cancer, participants were asked, “Based on what you know or believe, can drinking alcohol cause breast cancer/liver disease/the flu/harm to a fetus? Yes/no/don’t know”; only responses to the cancer item are reported here. Responses were dichotomized as 0 = *no/don’t know* and 1 = *yes;* 5 (0.6%) participants answered “prefer not to say”/missing and were excluded from these analyses.

### Knowledge of standard drinks in preferred beverage type

To assess knowledge of standard drink information, participants were asked to report the number of standard drinks in a container of their preferred beverage type. An image of a container of their preferred drink was shown on-screen, and the container label listed the volume in milliliters and the percentage alcohol by volume. The range of correct responses for the number of standard drinks was between 1.26 and 1.54 for beer, 4 and 6 for wine, 16 and 20 for distilled spirits, and 0.9 and 1.1 for ciders, which is 10% above and below the accurate number of standard drinks for each beverage type. Answers were dichotomized to 0 = *incorrect* and 1 = *correct;* 16 (1.9%) participants responded “prefer not to say”/missing and were excluded from these analyses.

### Knowledge of sex-specific daily and weekly low-risk drinking guideline limits

To assess knowledge of sex-specific daily and weekly recommended drink limits in Canada’s LRDG, participants were asked, “What is the daily (or weekly) limit of ‘standard drinks’ recommended for males/females (depending on identified sex) in Canada’s Low-Risk Drinking Guidelines?” The number of daily or weekly standard drinks was entered as an open-ended item. The range of correct responses for women was two standard drinks or less (0–2) per day and 10 standard drinks or less (0–10) per week, and for men it was three standard drinks or less (0–3) per day and 15 standard drinks or less (0–15) per week. Responses were dichotomized as 0 = *incorrect* and 1 = *correct;* 10 (1.2%) participants responded “prefer not to say”/missing and were excluded from these analyses.

### Support for alcohol warning labels with a health message, standard drink information, and low-risk drinking guideline limits

To assess support for AWLs on alcohol containers, participants were asked the degree to which they agree or disagree with the following: “Cans and bottles of alcoholic beverages should be labeled with warnings describing the link between alcohol and diseases, such as cancer”; “Cans and bottles of alcoholic beverages should be labeled with the number of standard drinks per container”; “Cans and bottles of alcoholic beverages should be labeled with low-risk drinking guidelines.” Responses for the three support measures included a 5-point Likert scale, which was dichotomized as 0 = *neutral/disagree/strongly disagree/don’t know* and 1 = *agree/strongly agree.* Eight participants (1.0%) for health messages and standard drink information and 10 participants (1.2%) for LRDG responded “prefer not to say”/missing and were excluded from these analyses.

### Sociodemographic and alcohol drinking characteristics

Sociodemographic measures included age, sex, ethnicity (White, Aboriginal/other, and unknown), highest level of education attained (completed high school or less, and more than high school, and unknown), and annual household income ($60,000, >$60,000, and unknown). (All amounts are in Canadian dollars.) Health literacy was measured using the Newest Vital Sign assessment tool (≤3, 4–6, and unknown); ≤1–3 correct responses represents limited/possibility of limited literacy, and 4–6 correct responses represents adequate literacy (Weiss et al., 2005). Pattern of alcohol consumption was measured using the quantity/frequency method (Heeb & Gmel, 2005). Participants were asked to indicate how often they drank alcoholic beverages in the past 6 months and how many drinks they usually drank per occasion. Responses were combined to provide a mean number of drinks per week and were categorized using Canada’s LRDG weekly limits (≤10/15 female/male per week, >10/15 female/male per week, and unknown) (Butt et al., 2011).

### Statistical analyses

Chi-square analyses (Cody & Smith, 1997) were conducted to assess differences in sociodemographic characteristics by site. To estimate predictors of knowledge that alcohol can cause cancer, of the correct number of standard drinks in a container, and of the correct daily/weekly LRDG limits, four separate multivariable logistic regression models were conducted. To estimate support for AWLs with a health message, standard drink information, and LRDG, three separate multivariable logistic regression models were conducted. Site as well as sociodemographic and alcohol drinking characteristics were entered as independent variables in all models. Adjusted odds ratios (AORs) and corresponding 95% confidence intervals (CIs) were estimated to quantify associations. Sociodemographic and alcohol drinking variables with “don’t know”/“prefer not to say”/missing responses were treated as a separate “unknown” category in the analyses, and AORs and CIs are not presented for this category. As per agreement with the local territorial government partners, ethnicity is included in the sample description and is controlled for in the analyses but is not reported in the results. All analyses were performed using SAS 9.3 (SAS Institute Inc., Cary, NC).

## Results

In total, 836 participants completed the baseline survey, with 505 (60.4%) in the intervention site and 331 (39.6%) in the comparison site (Table 1). A higher proportion of participants 45 years and older were in the intervention site compared with the comparison site (62.0% vs. 44.4%, *p* = .0001). There was also a significant difference in ethnicity, with a higher proportion of participants in the intervention than in the comparison site identifying as White (72.9% vs. 65.3%, *p* = .0005). A higher proportion of participants in the intervention site compared with the comparison site also reported alcohol consumption above the recommended weekly LRDG limits (18.8% vs. 15.4%, *p* = .0391). There were no other significant differences between the two sites by key sociodemographic characteristics. Overall, 400 (47.9%) participants identified as female, 623 (74.5%) had more than a high school education, 465 (55.6%) reported an annual household income of $60,000 or greater, and 322 (38.5%) participants had adequate health literacy.

**Table 1.**
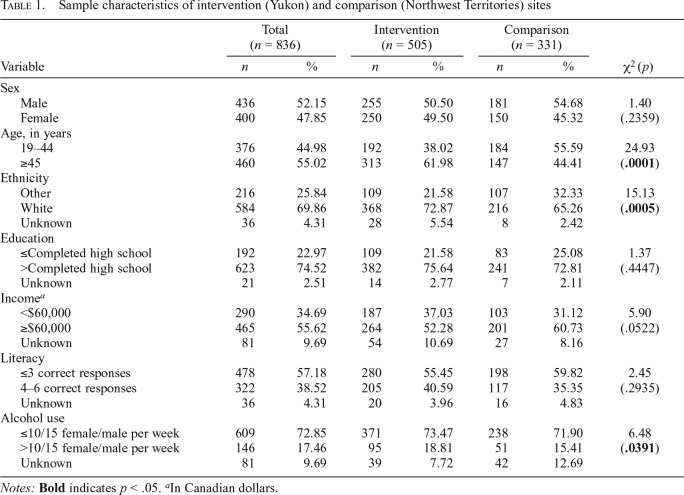
Sample characteristics of intervention (Yukon) and comparison (Northwest Territories) sites

Variable	Total (*n* = 836)		Intervention (*n* = 505)		Comparison (*n* = 331)		
	*n*	%	*n*	%	*n*	%	χ2 (*p*)
Sex							
Male	436	52.15	255	50.50	181	54.68	1.40
Female	400	47.85	250	49.50	150	45.32	(.2359)
Age, in years							
19–44	376	44.98	192	38.02	184	55.59	24.93
≥45	460	55.02	313	61.98	147	44.41	(**.0001**)
Ethnicity							
Other	216	25.84	109	21.58	107	32.33	15.13
White	584	69.86	368	72.87	216	65.26	(**.0005**)
Unknown	36	4.31	28	5.54	8	2.42	
Education							
≤Completed high school	192	22.97	109	21.58	83	25.08	1.37
>Completed high school	623	74.52	382	75.64	241	72.81	(.4447)
Unknown	21	2.51	14	2.77	7	2.11	
Income*^a^*							
<$60,000	290	34.69	187	37.03	103	31.12	5.90
≥$60,000	465	55.62	264	52.28	201	60.73	(.0522) 54
Unknown	81	9.69	54	10.69	27	8.16	
Literacy							
≤3 correct responses	478	57.18	280	55.45	198	59.82	2.45
4–6 correct responses	322	38.52	205	40.59	117	35.35	(.2935)
Unknown	36	4.31	20	3.96	16	4.83	
Alcohol use							
≤10/15 female/male per week	609	72.85	371	73.47	238	71.90	6.48
>10/15 female/male per week	146	17.46	95	18.81	51	15.41	(**.0391**)
Unknown	81	9.69	39	7.72	42	12.69	

*Notes:* **Bold** indicates *p* < .05.

^a^ In Canadian dollars.

### Knowledge that alcohol can cause cancer

Overall, 204 (24.5%) participants knew that drinking alcohol can cause cancer, with no significant differences between intervention and comparison sites (Table 2). Of those that knew alcohol causes cancer, 100 (23.1%) were male and 104 (26.1%) were female; there was no significant difference between men and women for this outcome (AOR = 1.18, 95% CI [0.86, 1.63]). Results of the multivariable logistic regression model indicated no significant differences in cancer knowledge across sociodemographic factors.

**Table 2.**
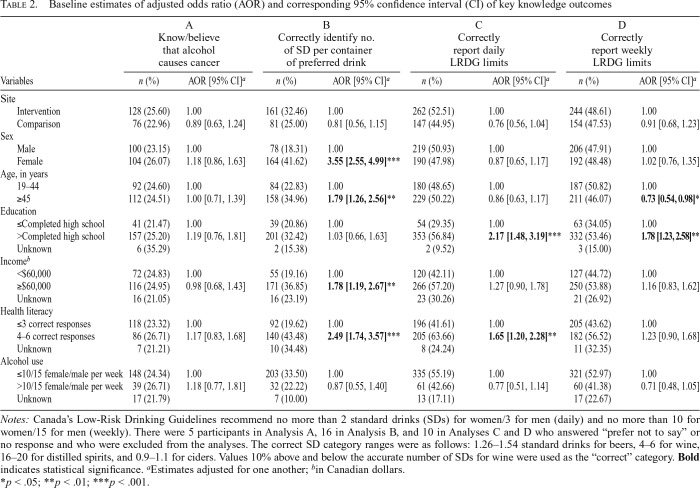
Baseline estimates of adjusted odds ratio (AOR) and corresponding 95% confidence interval (CI) of key knowledge outcomes

	A		B		C		D	
	Know/believe that alcohol causes cancer		Correctly identify no. of SD per container of preferred drink		Correctly report daily LRDG limits		Correctly report weekly LRDG limits	
Variables	*n* (%)	AOR [95% CI]^*a*^	*n* (%)	AOR [95% CI]*^a^*	*n* (%)	AOR [95% CI]*^a^*	*n* (%)	AOR [95% CI]*^a^*
Site								
Intervention	128 (25.60)	1.00	161 (32.46)	1.00	262 (52.51)	1.00	244 (48.61)	1.00
Comparison	76 (22.96)	0.89 [0.63, 1.24]	81 (25.00)	0.81 [0.56, 1.15]	147 (44.95)	0.76 [0.56, 1.04]	154 (47.53)	0.91 [0.68, 1.23]
Sex								
Male	100 (23.15)	1.00	78 (18.31)	1.00	219 (50.93)	1.00	206 (47.91)	1.00
Female	104 (26.07)	1.18 [0.86, 1.63]	164 (41.62)	3.55 [2.55, 4.99]***	190 (47.98)	0.87 [0.65, 1.17]	192 (48.48)	1.02 [0.76, 1.35]
Age, in years								
19–44	92 (24.60)	1.00	84 (22.83)	1.00	180 (48.65)	1.00	187 (50.82)	1.00
≥45	112 (24.51)	1.00 [0.71, 1.39]	158 (34.96)	1.79 [1.26, 2.56]**	229 (50.22)	0.86 [0.63, 1.17]	211 (46.07)	0.73 [0.54, 0.98]*
Education								
≤Completed high school	41 (21.47)	1.00	39 (20.86)	1.00	54 (29.35)	1.00	63 (34.05)	1.00
>Completed high school	157 (25.20)	1.19 [0.76, 1.81]	201 (32.42)	1.03 [0.66, 1.63]	353 (56.84)	2.17 [1.48, 3.19]***	332 (53.46)	1.78[1.23,2.58]**
Unknown	6 (35.29)		2 (15.38)		2 (9.52)		3 (15.00)	
Income*^b^*								
<$60,000	72 (24.83)	1.00	55 (19.16)	1.00	120 (42.11)	1.00	127 (44.72)	1.00
≥$60,000	116 (24.95)	0.98 [0.68, 1.43]	171 (36.85)	1.78 [1.19, 2.67]**	266 (57.20)	1.27 [0.90, 1.78]	250 (53.88)	1.16 [0.83, 1.62]
Unknown	16 (21.05)		16 (23.19)		23 (30.26)		21 (26.92)	
Health literacy								
≤3 correct responses	118 (23.32)	1.00	92 (19.62)	1.00	196 (41.61)	1.00	205 (43.62)	1.00
4–6 correct responses	86 (26.71)	1.17 [0.83, 1.68]	140 (43.48)	2.49 [1.74, 3.57]***	205 (63.66)	1.65 [1.20, 2.28]**	182 (56.52)	1.23 [0.90, 1.68]
Unknown	7 (21.21)		10 (34.48)		8 (24.24)		11 (32.35)	
Alcohol use							
≤10/15 female/male per week	148 (24.34)	1.00	203 (33.50)	1.00	335 (55.19)	1.00	321 (52.97)	1.00
>10/15 female/male per week	39 (26.71)	1.18 [0.77, 1.81]	32 (22.22)	0.87 [0.55, 1.40]	61 (42.66)	0.77 [0.51, 1.14]	60 (41.38)	0.71 [0.48, 1.05]
Unknown	17 (21.79)		7 (10.00)		13 (17.11)		17 (22.67)	

*Notes:* Canada’s Low-Risk Drinking Guidelines recommend no more than 2 standard drinks (SDs) for women/3 for men (daily) and no more than 10 for women/15 for men (weekly). There were 5 participants in Analysis A, 16 in Analysis B, and 10 in Analyses C and D who answered “prefer not to say” or no response and who were excluded from the analyses. The correct SD category ranges were as follows: 1.26–1.54 standard drinks for beers, 4–6 for wine, 16–20 for distilled spirits, and 0.9–1.1 for ciders. Values 10% above and below the accurate number of SDs for wine were used as the “correct” category. **Bold** indicates statistical significance.

^a^ Estimates adjusted for one another;

^b^ in Canadian dollars.

* *p* < .05;

** *p* < .01;

*** *p* < .001.

### Knowledge of standard drinks in preferred beverage type

A total of 242 (29.5%) participants correctly reported the number of standard drinks in a container of their preferred beverage type, with no significant differences between sites (Table 2). Regression results indicate that women (AOR = 3.55, 95% CI [2.55, 4.99]), those 45 years and older (AOR = 1.79, 95% CI [1.26, 2.56]), those with an annual household income of $60,000 or greater (AOR = 1.78, 95% CI [1.19, 2.67]), and those with adequate health literacy (AOR = 2.49, 95% CI [1.74, 3.57]) had greater odds, compared with the referent group, of correctly reporting the number of standard drinks in a container of their preferred beverage type.

### Knowledge of sex-specific daily and weekly LRDG limits

Overall, 409 (49.5%) participants were able to correctly report the sex-specific daily LRDG limits, and 398 (48.2%) were able to report the sex-specific weekly LRDG limits (Table 2); there were no significant differences between sites. Regression results indicate that participants who reported having more than a high school education (AOR = 2.17, 95% CI [1.48, 3.19]) and with adequate health literacy (AOR = 1.65, 95% CI [1.20, 2.28]) had greater odds, compared with the referent group, of correctly reporting the daily LRDG limits. Participants 45 years and older (AOR = 0.73, 95% CI [0.54, 0.98]) had lower odds, compared with the referent group, of correctly reporting the weekly LRDG limits, and those with more than a high school education (AOR = 1.78, 95% CI [1.23, 2.58]) had greater odds, compared with the referent group, of correctly reporting the weekly LRDG limits.

### Support for alcohol warning labels with health message, standard drink information, and low-risk drinking guideline limits

In total, 463 (55.9%) participants agreed or strongly agreed that alcohol containers should be labeled with AWLs including a health warning, 426 (51.4%) participants agreed or strongly agreed containers should be labeled with standard drink information, and 320 (38.3%) participants agreed or strongly agreed containers should be labeled with LRDG limits; there were no significant differences between intervention and comparison sites. Regression results indicate that participants who identified as female (AOR = 1.45, 95% CI [1.09, 1.92]) and with adequate health literacy (AOR = 1.71, 95% CI [1.25, 2.36]) had greater odds, compared with the referent group, of supporting labeling containers with a health warning (Table 3). Similarly, participants who identified as female (AOR = 1.70, 95% CI [1.27, 2.27]), those with more than a high school education (AOR = 1.44, 95% CI [1.00, 2.03]), and those with adequate health literacy (AOR = 2.02, 95% CI [1.47, 2.78]) had greater odds, compared with the referent group, of supporting labeling containers with standard drink information. Participants who reported consuming above the weekly LRDG limits (AOR = 0.60, 95% CI [0.40, 0.88]) had lower odds, compared with the referent group, of supporting labeling containers with standard drink information (Table 3). Similarly, participants who identified as female (AOR = 1.55, 95% CI [1.15, 2.07]), those 45 years and older (AOR = 1.49, 95% CI [1.09, 2.02]), those with more than a high school education (AOR = 1.75, 95% CI [1.18, 2.58]), and those with adequate health literacy (AOR = 1.55, 95% CI [1.12, 2.14]) had greater odds, compared with the referent group, of supporting labeling containers with LRDG limits. Participants with an annual household income of $60,000 or greater (AOR = 0.64, 95% CI [0.45, 0.90]) and who reported consuming above the weekly LRDG limits (AOR = 0.57, 95% CI [0.38, 0.87]) had lower odds, compared with the referent group, of supporting labeling containers with LRDG limits (Table 3).

**Table 3.**
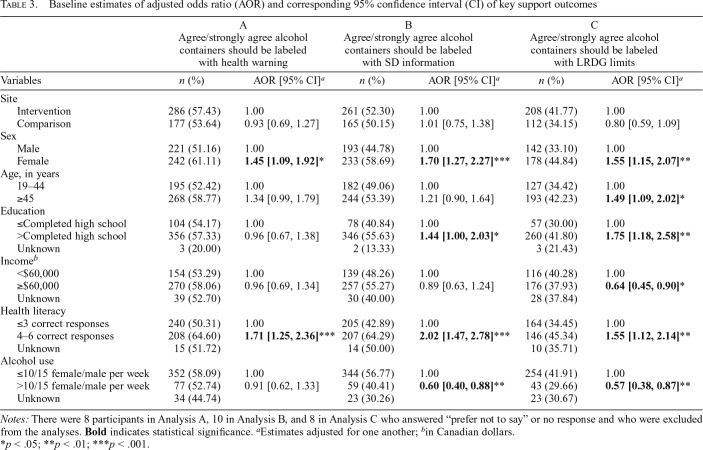
Baseline estimates of adjusted odds ratio (AOR) and corresponding 95% confidence interval (CI) of key support outcomes

	A		B		C	
	Agree/strongly agree alcohol containers should be labeled with health warning		Agree/strongly agree alcohol containers should be labeled with SD information		Agree/strongly agree alcohol containers should be labeled with LRDG limits	
Variables	*n* (%)	AOR [95% CI]*^a^*	*n* (%)	AOR [95% CI]*^a^*	*n* (%)	AOR [95% CI]*^a^*
Site						
Intervention	286 (57.43)	1.00	261 (52.30)	1.00	208 (41.77)	1.00
Comparison	177 (53.64)	0.93 [0.69, 1.27]	165 (50.15)	1.01 [0.75, 1.38]	112 (34.15)	0.80 [0.59, 1.09]
Sex						
Male	221 (51.16)	1.00	193 (44.78)	1.00	142 (33.10)	1.00
Female	242 (61.11)	1.45 [1.09, 1.92]*	233 (58.69)	1.70 [1.27, 2.27]***	178 (44.84)	1.55 [1.15, 2.07]**
Age, in years						
19–44	195 (52.42)	1.00	182 (49.06)	1.00	127 (34.42)	1.00
≥45	268 (58.77)	1.34 [0.99, 1.79]	244 (53.39)	1.21 [0.90, 1.64]	193 (42.23)	1.49 [1.09, 2.02]*
Education						
≤Completed high school	104 (54.17)	1.00	78 (40.84)	1.00	57 (30.00)	1.00
>Completed high school	356 (57.33)	0.96 [0.67, 1.38]	346 (55.63)	1.44 [1.00, 2.03]*	260 (41.80)	1.75 [1.18, 2.58]**
Unknown	3 (20.00)		2 (13.33)		3 (21.43)	
Income*^b^*						
<$60,000	154 (53.29)	1.00	139 (48.26)	1.00	116 (40.28)	1.00
≥$60,000	270 (58.06)	0.96 [0.69, 1.34]	257 (55.27)	0.89 [0.63, 1.24]	176 (37.93)	0.64 [0.45, 0.90]*
Unknown	39 (52.70)		30 (40.00)		28 (37.84)	
Health literacy						
≤3 correct responses	240 (50.31)	1.00	205 (42.89)	1.00	164 (34.45)	1.00
4–6 correct responses	208 (64.60)	1.71 [1.25, 2.36]***	207 (64.29)	2.02 [1.47, 2.78]***	146 (45.34)	1.55 [1.12, 2.14]**
Unknown	15 (51.72)		14 (50.00)		10 (35.71)	
Alcohol use							
≤10/15 female/male per week	352 (58.09)	1.00	344 (56.77)	1.00	254 (41.91)	1.00
>10/15 female/male per week	77 (52.74)	0.91 [0.62, 1.33]	59 (40.41)	0.60 [0.40, 0.88]**	43 (29.66)	0.57 [0.38, 0.87]**
Unknown	34 (44.74)		23 (30.26)		23 (30.67)	

*Notes:* There were 8 participants in Analysis A, 10 in Analysis B, and 8 in Analysis C who answered “prefer not to say” or no response and who were excluded from the analyses. **Bold** indicates statistical significance.

^a^ Estimates adjusted for one another;

^b^ in Canadian dollars.

* *p* < .05;

** *p* < .01;

*** *p* < .001.

## Discussion

This study assessed baseline knowledge of alcohol-related health information and support for AWLs as well as the associations between these outcomes and key sociodemographic characteristics among liquor store patrons in two northern Canadian territories. Overall, there were no significant differences in the main outcomes between participants in Whitehorse and Yellowknife at baseline, indicating that the cities were suitably matched as intervention and comparison sites. Further, this population had relatively low levels of alcohol-related knowledge, which provides justification for the broader study testing the impact of labels with messages related to alcohol and cancer risk, national drinking guidelines, and standard drink information across jurisdictions.

Roughly a quarter of the sample knew that alcohol can cause cancer, which is comparable to the relatively low awareness levels found in previous national and international studies (Buykx et al., 2016; Canadian Cancer Society, 2015; Rundle-Thiele et al., 2013; Scheideler & Klein, 2018; Wiseman & Klein, 2019) and which is anticipated given ongoing alcohol industry efforts to keep this information from the public (Petticrew et al., 2018a, 2018b; Vallance et al., 2020a, 2020b). There were no sociodemographic factors associated with knowing that alcohol is a carcinogen, suggesting that, regardless of age, sex, socioeconomic status, or pattern of alcohol consumption, awareness of this serious alcohol-related health risk remains consistently unknown. Considering the large proportion of Canadians who have indicated that knowledge of alcohol-cancer risk would decrease their consumption (Canadian Cancer Society, 2015), this information is important for consumers to make more informed choices and could potentially contribute to a shift in drinking patterns.

Similar to the findings of previous Canadian studies (McNally et al., 2019; Osiowy et al., 2015; Hobin et al., 2018), less than a third of the overall sample was able to correctly estimate the number of standard drinks in a container of their preferred alcoholic beverage when only volume and percentage alcohol-by-volume information were presented on the label. Women and older participants, as well as those with higher income and health literacy levels, were better able to calculate standard drinks using this limited label information. This outcome further highlights that presenting only percentage alcohol-by-volume information on alcohol labels may unduly disempower more vulnerable and higher consuming groups from accurately tracking their consumption and preventing or reducing harms.

Also consistent with previous research (McNally et al., 2019; Osiowy et al., 2015; Hobin et al., 2018), a comparably low proportion of participants—less than half overall—accurately reported the sex-specific daily and weekly limits recommended in Canada’s LRDG. Knowledge of both sets of drink limits was greater among those with higher education levels, and for the daily limits it was greater among those also with higher health literacy—again suggesting that there is a need for more consistent and accessible exposure to national guidelines. Taken together, these results support previous recommendations (Osiowy et al., 2015; Hobin et al., 2018; Wettlaufer, 2018) to provide both standard drink and sex-specific drink limit information on alcohol container labels to improve all consumers’ ability to estimate not only their total consumption but also their consumption in relation to national drinking guidelines. Providing this combination in an accessible format on alcohol containers would expose a broader range of alcohol consumers—including high-volume drinkers (Greenfield, 1997)—to this information.

Broad support for labeling alcohol containers with a health warning such as cancer risk and standard drink and LRDG information was moderate among this population, with more than half of drinkers supporting labels with a health warning and standard drink information. These findings are in line with Canadian and international research showing that the public supports providing this type of label information—and especially cancer warnings—on alcohol containers (Bates et al., 2018; Buykx et al., 2015; Hobin et al., 2018; Miller et al., 2016; Osiowy et al., 2015; Pettigrew et al., 2014; Thomson et al., 2012; Vallance et al., 2018). Overall support for labeling alcohol containers with the three different types of messages was highest among women and those with higher health literacy levels, which are similar characteristics noted in support for most alcohol policies (Bates et al., 2018; Buykx et al., 2016; Li et al., 2017; Moskalewicz et al., 2013; Pechey et al., 2014; Rundle-Thiele, 2013). Despite the consistent acceptability of labels among this sample and across different jurisdictions and population groups, implementation of evidence-informed labels remains low internationally. This discrepancy points to other barriers to their introduction—including commercial vested interests of keeping consumers in the dark about alcohol-related harms such as cancer risk and lobbying by powerful alcohol industry groups—rather than a lack of public support (Bhattacharya et al., 2018; Casswell et al., 2016; Connor, 2017; Vallance et al., 2020b).

Reporting alcohol consumption levels above the recommended weekly LRDG limits was associated with lower levels of support for both standard drink and LRDG labels, which is consistent with previous research finding that those with higher consumption levels are less supportive of alcohol policies (Bates et al., 2018; Li et al., 2017; Macdonald et al., 2011; Moskalewicz et al., 2013; Pechey et al., 2014; Wilkinson et al., 2009). Interestingly, higher alcohol consumption was not associated with a lower likelihood of supporting labels with a health warning such as cancer risk—which may suggest that consumers would not object to these types of labels regardless of their alcohol consumption patterns. Although displaying LRDG information on labels received less support in this sample and elsewhere (Li et al., 2017), the potentially synergistic effect on consumers’ ability to more accurately monitor their consumption when combined with standard drink measurements warrants their inclusion (Bates et al., 2018).

### Limitations

Study limitations include a low response rate common to this type of intercept recruitment technique (Hobin et al., 2017; Schneider, 2013; Wiggers et al., 2018) and participant recruitment from liquor stores in the city centers using nonprobability methods. The sample was therefore not representative of site populations, which limits generalizability. However, the distributions of age, sex, and ethnicity are similar to those in the sample of drinkers in the 2014 Canadian Community Health Survey and can thus be considered broadly representative of people who drink alcohol in Yukon and Northwest Territories. The use of self-report surveys may also be subject to response bias. In addition, only one prompted measure specific to breast cancer was used to test knowledge of alcohol’s carcinogenicity. Future research could include both prompted and unprompted measures assessing knowledge of risk for multiple cancer types.

### Conclusion

This study identified low baseline levels of knowledge of alcohol-related harm, such as cancer risk, limited ability to calculate number of standard drinks in containers using currently mandated labeling information, and low knowledge of Canada’s LRDG limits. There was support for AWLs that included a health message such as cancer risk, standard drink information, and national low-risk drinking guidelines. Implementation of evidence-based AWLs is warranted and is likely to receive public support as a tool to increase awareness of alcohol related-risks and to support Canadian consumers in the North and elsewhere to make more informed and safer alcohol choices.

## Acknowledgments

The authors acknowledge all of our RAs who helped with data collections, as well as the liquor control boards, health and social services, and especially the community partners in Yukon and Northwest Territories for their commitment and support in developing and executing this research. Special thanks also go to Mark Petticrew and Melanie Wakefield for their expertise and guidance.
